# When disease travels downstream: The effect of intravesical recurrences in patients treated for upper tract urothelial carcinoma

**DOI:** 10.1002/bco2.70225

**Published:** 2026-06-29

**Authors:** Orlane J. A. Figaroa, Sanne J. Jonker, Felix J. Fris, Guido M. Kamphuis, Adriaan D. Bins, R. Jeroen A. van Moorselaar, Joyce Baard

**Affiliations:** ^1^ Department of Urology, Amsterdam UMC University of Amsterdam Amsterdam The Netherlands; ^2^ Department of Medical Oncology, Amsterdam UMC University of Amsterdam Amsterdam The Netherlands; ^3^ Cancer Center Amsterdam Amsterdam UMC Amsterdam The Netherlands; ^4^ Department of Urology Amsterdam UMC, VU Amsterdam The Netherlands; ^5^ Faculty of medicine University of Amsterdam Amsterdam The Netherlands

**Keywords:** intravesical recurrences, radial nephroureterectomy, survival outcomes, upper tract urothelial cancer, ureteroscopy

## Abstract

**Background:**

Treatment of upper tract urothelial carcinoma (UTUC) depends on risk stratification and tumour characteristics. Kidney‐sparing surgery (KSS) is preferred for low‐risk disease, whereas radical nephroureterectomy (RNU) is the standard for high‐risk UTUC. Intravesical recurrences (IVR) are common after both treatment modalities, but their impact on survival remains unclear.

**Objective:**

To assess the IVR rate and cumulative hazard following UTUC treatment and oncological outcomes based on the presence of IVR during follow‐up. Second, the relation between UTUC and IVR tumour grade in patients treated with endoscopic KSS (eKSS).

**Methods:**

A single‐centre study, including non‐metastatic UTUC patients treated between 2010 and 2023. Analysis was performed in a cohort of patients without a history of bladder cancer.

**Results:**

In total 164 patients were selected; 85 treated with eKSS, 79 by RNU. Overall, 91 patients (55%) developed an IVR, 53 (62%) after eKSS and 38 (48%) after RNU, during a median follow‐up of 33 months (IQR 11–72). eKSS‐treated patients showed a significantly higher cumulative hazard for IVR (HR 0.6, 95% CI 0.42–0.95, *p* = 0.02). The CSS and MFS were comparable between patients with or without IVR during follow‐up. In patients treated by eKSS, we found IVR upgrading in 24% of patients treated with eKSS.

**Conclusions:**

Patients treated by eKSS showed a higher cumulative hazard of IVR, without an impact on oncological outcomes. These findings support the use of kidney‐sparing approaches in well‐selected cases and highlight the need for proper follow‐up including the bladder and evaluate IVR preventive measures as intravesical instillations.

## 1 INTRODUCTION

Upper tract urothelial carcinoma (UTUC) is a rare malignancy. Management of primary, non‐metastatic UTUC is guided by risk stratification according to the European Association of Urology (EAU) guidelines; patients with low‐risk disease can be offered kidney‐sparing surgery (KSS) by ureteroscopy (URS) with laser ablation (endoscopic KSS [eKSS]) or segmental ureter resection (SUR) in case of ureter tumours. Radical nephroureterectomy (RNU) is the preferred treatment for patients with high‐risk disease.^1^ In well‐selected low‐risk patients, eKSS provides comparable oncological and survival outcomes (SOs) to RNU but requires stringent surveillance due to the higher risk of recurrences.^2^ Careful follow‐up is indicated to detect both upper tract and intravesical recurrences (IVR). Surveillance modalities consist of cytology, cystoscopy, imaging by CT‐urography and URS to allow direct inspection of the upper urinary tract. Follow‐up regimens should be tailored to initial treatment, risk stratification and tumour characteristics as grade of recurrences in both upper and lower tract; however, detailed evidence‐based follow‐up protocols are lacking.^3^, ^4^

IVRs are common following the management of UTUC with reported rates ranging from 22% to 47% after RNU.^5^, ^6^ Only one meta‐analysis reports on IVR risk following eKSS alone, with an reported incidence of 23.7%.^7^ Theories for IVR pathogenesis include field cancerization and intra‐luminal seeding.^8^, ^9^ Intravesical instillations (IVIs) with agents such as mitomycin are recommended by the EAU guidelines post‐RNU, based on evidence from randomised trials and meta‐analyses reporting a reduction in early bladder recurrence in patients without a history of bladder cancer.^10^, ^11^, ^12^, ^13^ However, no evidence exist on the efficacy of IVIs post‐eKSS as only patients post‐RNU were included in the existing RCTs. While SOs following eKSS and RNU have been reported, the impact of IVR on survival after eKSS is not well established.^14^

In this study, we analyse IVR in UTUC patients treated with RNU or eKSS, evaluate oncological outcomes based on IVR during follow‐up and compare IVR tumour grade with the primary upper‐tract tumour after eKSS.

## 2 SUBJECTS AND METHODS

### 2.1 Objectives

Primary objectives were to assess IVR after RNU and eKSS and to evaluate oncological outcomes (CSS, OS, IVR‐FS, MFS) based on the presence of IVR during follow‐up. Secondary, we analysed the cumulative hazard of IVR after both treatments and compared IVR tumour grade with the primary UTUC, focusing on upgrading in the eKSS group.

### 2.2 Patient population and Selection

This single‐centre cohort included adults treated for non‐metastatic UTUC by eKSS (URS laser ablation) or RNU at Amsterdam UMC between 2010 and 2023. Exclusion criteria were metastatic disease, synchronous bilateral UTUC, segmental ureteral resection, percutaneous tumour resection, and UTUC in transplant kidneys. Patients were identified through our institutional UTUC database. Diagnostic workup included cystoscopy, CT urography, cytology and/or URS with biopsy. Treatment allocation was based on tumour grade, technical feasibility, renal function, and patient preference.

### 2.3 Data Collection and Follow‐up

Encrypted data included baseline patient and tumour characteristics, histopathology and follow‐up outcomes. Preoperative CT scans assessed tumour location, focality, invasion, nodal status and hydronephrosis. Tumour grade was determined by histopathology or, if unavailable, by cytology; stage was based on RNU specimen or imaging. IVI was given post‐RNU per EAU guidelines, and since March 2023, also post‐eKSS per local protocol. Follow‐up data included IVR, histopathology, metastasis and subsequent treatments. IVR was defined as macroscopic recurrence on cystoscopy and managed per EAU bladder cancer guidelines.^3^, ^4^

### 2.4 Statistical analysis

Patient demographics and tumour characteristics were analysed using percentage and interquartile range (IQR). Nelson–Aalen was used to visualise the cumulative hazard for development of IVR over time. To evaluate the hazard ratio for IVR, an Anderson–Gill analysis was used, a cox‐based regression model to analyse recurrent events, since standard cox‐regression models only consider the first event, and therefore leads in loss of valuable information. Different censoring criteria were used for each outcome measure: OS at the final documented patient contact; IVRFS for last URS without recurrence; CSS at the last contact without disease progression evidence; MFS at the most recent imaging showing no metastatic disease.

To visualise the relation between the initial UTUC grade and the tumour grade of the IVR, a Sankey diagram was made. To minimise the impact of a significant confounding factor on the development of IVR during follow‐up, all analyses were performed on patients without a history of bladder cancer. For the analysis, we used SPSS Version 26.0 (SPSS Inc.) and R‐studio Version 4.3 (R Foundation for Statistical Analysis).

## 3 RESULTS

A total of 164 patients were selected based on the selection criteria, of whom 85 underwent eKSS and 79 underwent RNU between January 2010 and December 2023. The majority were male (68%), with a median age of 68 years. High‐grade primary tumours were observed in nine patients (11%) and 45 patients (57%) in the eKSS and RNU groups, respectively. Postoperatively, 9 (11%) IVIs were administered post‐eKSS (from 2023) and 51 (65%) post‐RNU. In the remaining cases post‐RNU, IVI was withheld due to intra‐ or post‐operative signs suggestive of bladder perforation or based on patient preference. Overall, an IVR was detected in 91 patients (55%), 53 (62%) in the eKSS group and 38 (48%) following RNU. During a median follow‐up period of 33 months (IQR 11–72). A previous medical history of bladder cancer was present in 34% in the eKSS group and 19% in the RNU group. These patients were excluded in further survival analysis. Patient and tumour characteristics, stratified by treatment group, are presented in Table 1.

**TABLE 1:** Overview of patient demographics and tumour characteristics in UTUC patients treated by eKSS versus RNU.

Demographics	Overall	eKSS	RNU	*p* value
Participants	164	85	79	
Gender (male) (*n*, %)	112	61 (71%)	51 (65%)	0.32
Age (mean, IQR)	68 (60–77)	67 (58–77)	70 (64–78)	0.10
BMI (mean, IQR)	26 (23–29)	27 (24–29)	26 (23–30)	0.23
ASA‐score				
ASA‐1 (*n*, %)	17	7 (8%)	10 (13%)	0.35
ASA‐2 (*n*, %)	110	62 (72%)	48 (61%)	0.097
ASA‐3 (*n*, %)	37	16 (19%)	31 (27%)	0.24
ASA‐4 (*n*, %)	‐	‐	‐	
Preoperative eGFR				
<30 (*n*, %)	5	3 (4%)	2 (3%)	0.69
30–60 (*n*, %)	55	19 (22%)	36 (46%)	0.002
>60 (*n*, %)	94	57 (67%)	37 (47%)	0.004
Missing (*n*, %)	10	6 (7%)	4 (5%)	
Hydronephrosis (*n*, %)	43	10 (12%)	33 (42%)	<0.001
History of smoking (*n*, %)	130	68 (80%)	62 (79%)	0.81
History of malignancy (*n*, %)	77	42 (49%)	35 (44%)	0.51
UTUC (*n*, %)	6	6 (7%)	‐	0.02
Bladder cancer (*n*, %)	44	29 (34%)	15 (19%)	0.03
LG (*n*, %)	15	13 (15%)	2 (3%)	0.005
HG (*n*, %)	23	14 (17%)	9 (11%)	0.38
Missing (*n*, %)	6	2 (2%)	4 (5%)	
Tumour side (right) (*n*, %)	81	39 (46%)	42 (53%)	0.35
Focality (unifocal/multifocal) (*n*, %)	96/68	54/31 (64%/37%)	42/37 (53%/47%)	0.18
Tumour location				
Uretral (*n*, %)	75	39 (46%)	36 (46%)	0.97
Pyelum (*n*, %)	89	46 (54%)	43 (43%)	0.97
Tumour size (diameter ≤2 mm/>2 mm) (*n*, %)	67/92	44(52%)/40(47%)	23(29%)/52(66%)	0.006
Missing (*n*, %)	5	1 (1%)	4 (5%)	
dURS (*n*, %)	155	85 (100%)	70 (89%)	0.001
Biopsy during dURS (*n*, %)	148	84 (99%)	64 (81%)	<0.001
Grade (LG/HG) (*n*, %)	108/54	75/9 (88%/9%)	33/45 (42%/57%)	<0.001
Missing (*n*, %)	2	1 (1%)	1 (1%)	
T stage				
Ta (*n*, %)	112	77 (90%)	35 (44%)	<0.001
T1 (*n*, %)	13	2 (2%)	11 (14%)	0.009
Tis (*n*, %)	1	‐	1 (1%)	
≥T2 (*n*, %)	32	‐	32 (17%)	<0.001
Missing (*n*, %)	5	5 (6%)	‐	
IVI (*n*, %)	60	9 (11%)	51 (65%)	<0.001
IVR (*n*, %)	91	53 (62%)	38 (48%)	0.067
Follow‐up in months (mean, IQR)	33 (11–72)	47 (19–79)	26 (7–58)	0.037

Abbreviations: dURS, diagnostic ureteroscopy; eKSS, endoscopic kidney‐sparing surgery; HG, high‐grade; IVI, intravesical installations; IVR, intravesical recurrences; LG, low grade; RNU, radical nephroureterectomy; UTUC, upper tract urothelial carcinoma.

### 3.1 Intravesical recurrence during follow‐up after RNU versus eKSS

**FIGURE 1:**
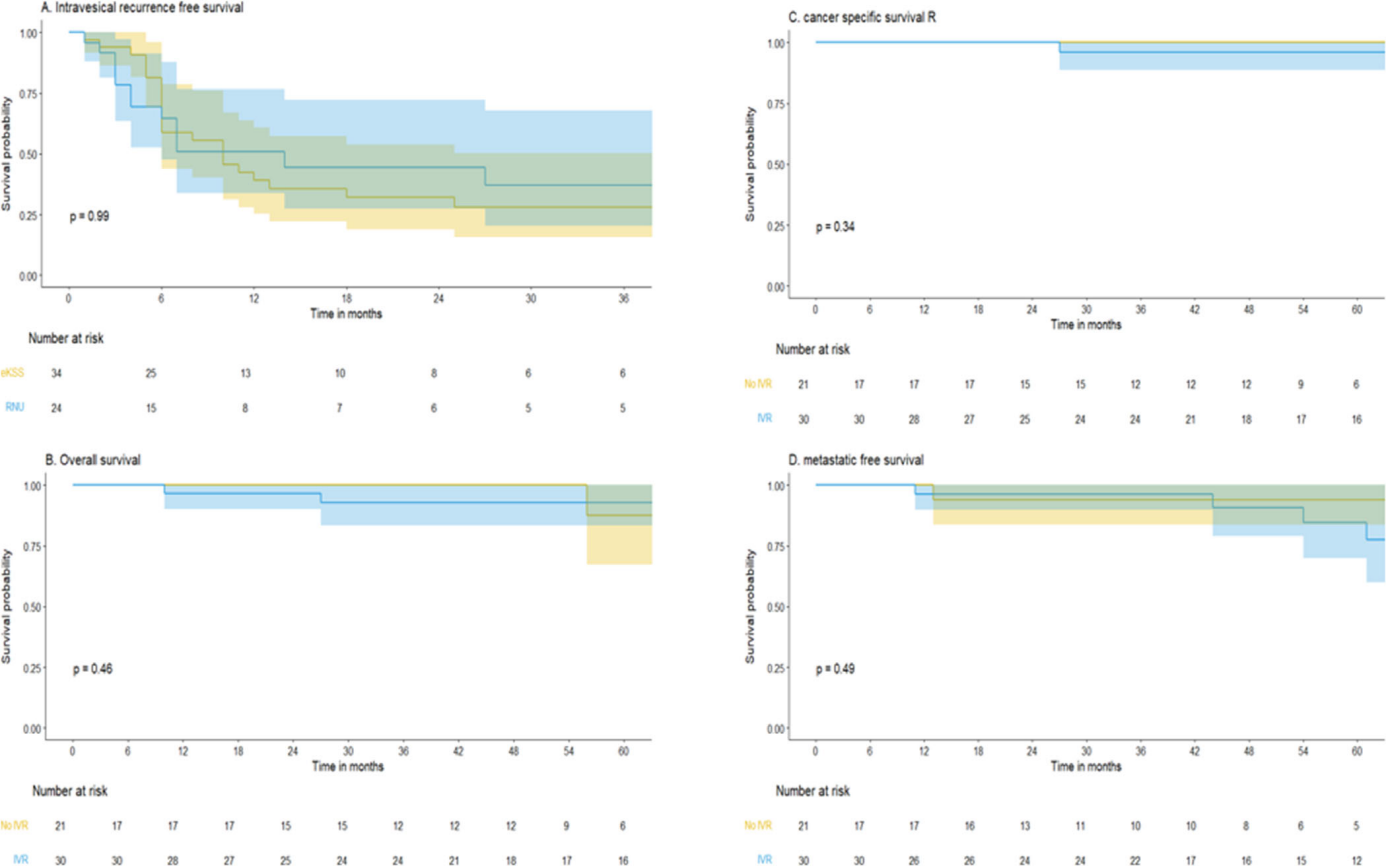
Kaplan–Meier of (A) the intravesical recurrences (IVR)‐FS in patients without a history of bladder cancer treated by radical nephroureterectomy (RNU) versus endoscopic kidney‐sparing surgery (eKSS); (B) CSS in patients without a history of bladder cancer treated by eKSS (intravesical recurrences [IVR] vs. no‐IVR); (C) OS in patients without a history of bladder cancer treated by eKSS (IVR vs. no‐IVR); (D) MFS in patients without a history of bladder cancer treated by eKSS (IVR vs. no‐IVR).

**FIGURE 2:**
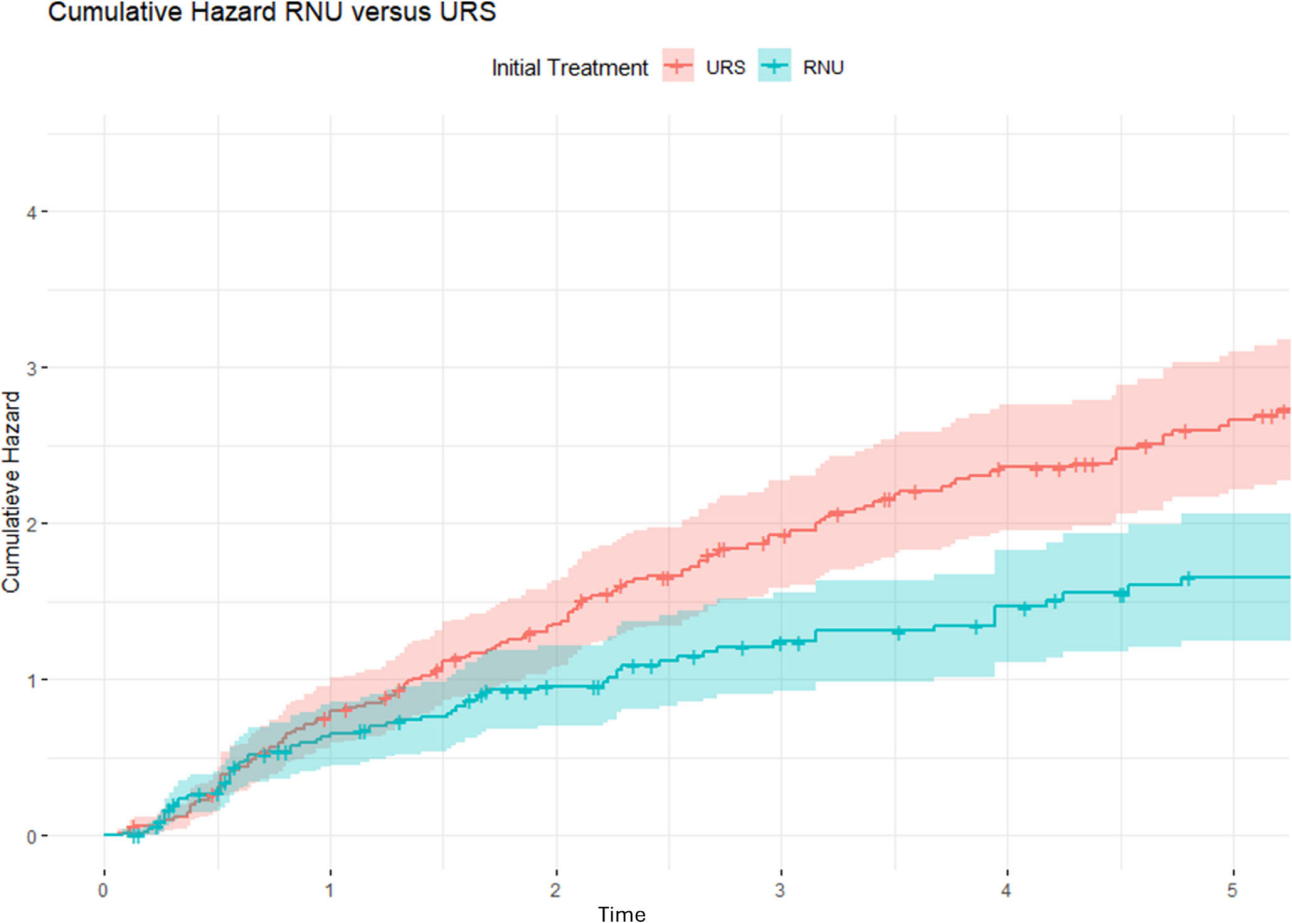
Nelson–Aalen plot for the cumulative hazard for intravesical recurrences (IVR) in patients treated by radical nephroureterectomy (RNU) versus ureteroscopy (URS) in time in years.

The 1 and 2‐year IVR‐free survival rates were 40% and 32% post‐eKSS group and 50% and 45% post‐RNU, respectively (Figure 1A), with a hazard ratio of 0.6 (95% CI 0.42–0.95) for IVR development during follow‐up. The median IVR‐FS was 10 months (IQR 6–14) post‐eKSS and 14 (IQR 6–18) months post‐RNU. The cumulative hazard for developing IVR over time is plotted in Figure 2 using the Nelson–Aalen method. Comparing both treatment modalities, treatment with RNU was associated with a significantly lower risk of developing IVR when considering all recurrences during this period (*p* = 0.02) using the Andersen and Gill analysis for recurrent events.

### 3.2 Oncological outcomes in patients after eKSS

In eKSS‐treated patients, SOs were compared based on the development of IVR. Patients without IVR had 1‐, 2‐ and 5‐year CSS rates of 100%, 100% and 100%, versus 100%, 100% and 97% in those with IVR (*p* = 0.34). Corresponding OS rates of 100%, 100% and 88% compared with 97%, 97% and 93%, respectively (*p* = 0.46), and MFS rates of 100%, 95% and 95% in patients without IVR, versus 97%, 97% and 85% in those with IVR (*p* = 0.49). Kaplan–Meier curves for these outcomes are shown in Figure 1B–D.

### 3.3 Oncological outcomes in patients after RNU

**FIGURE 3:**
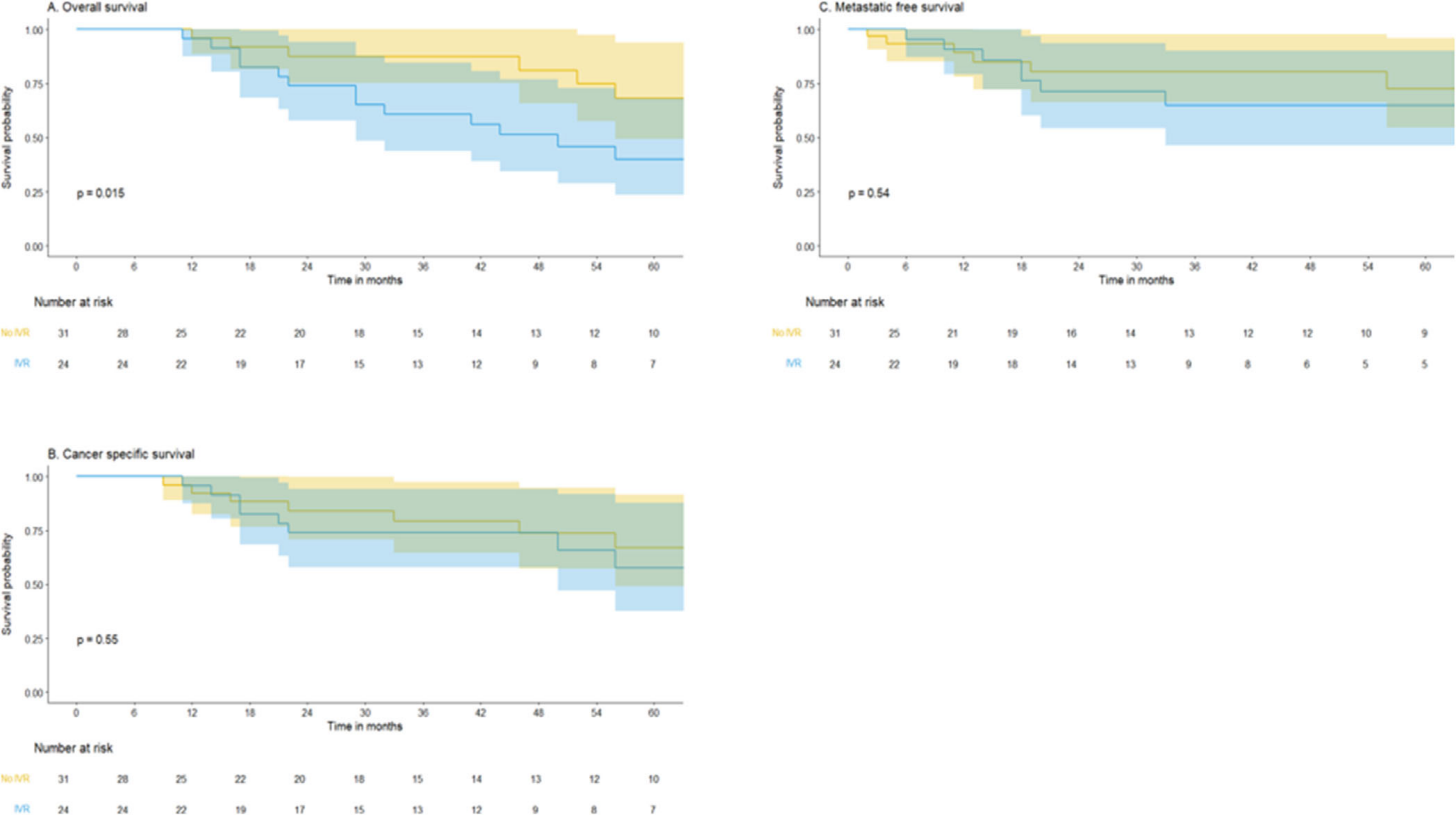
Kaplan–Meier of (A) CSS in patients without a history of bladder cancer treated by RNU (intravesical recurrences [IVR] vs. no‐IVR); (B) OS in patients without a history of bladder cancer treated by radical nephroureterectomy (RNU) (IVR vs. no‐IVR); (C) MFS in patients without a history of bladder cancer treated by RNU (IVR vs. no‐IVR).

In RNU‐treated patients, SOs based on the presence of an IVR did not significantly differ for CSS and MFS. Patients without IVR demonstrated 1‐, 2‐ and 5‐year CSS rates of 92%, 84% and 67%, compared with 96%, 74% and 56% in those who developed IVR (*p* = 0.55). MFS rates were 89%, 81% and 72% in patients without IVR, compared with 91%, 71% and 65% in patients with IVR (*p* = 0.54). However, we did observe a significantly lower OS for patients with an IVR in follow‐up (*p* = 0.015). The corresponding OS rates were 96%, 88% and 68% in patients without IVR, versus 96%, 74% and 40% in those with IVR. Kaplan–Meier curves for these outcomes are shown in Figure 3.

### 3.4 Tumour grading

We evaluated the relation between the tumour grade of the initial UTUC and the grade of subsequent IVRs. For this analysis, only patients post‐eKSS without a prior history of bladder cancer were selected. Among patients with low‐grade (LG) UTUC, a total of 21 IVRs occurred, of which 16 (76%) were LG and five (24%) were high‐grade (HG). In the HG‐UTUC group, 12 IVRs were observed, of which 10 (83%) were HG. Of the patients who developed a first LG‐IVR, 10 experienced a second IVR, nine (90%) of which were LG. Seven of these patients had a third recurrence, five of whom (71%) remained LG. The Sankey plot illustrates the distribution of tumour grades across successive recurrences. (Figure S1).

## 4 DISCUSSION

In this retrospective cohort study, we evaluated the incidence, characteristics and oncological outcomes based on IVR following treatment for UTUC. We analysed the cumulative hazard of IVR during follow‐up and examined the relation between primary UTUC tumour grade and subsequent IVR grade.

No statistically significant difference in IVR‐FS was observed between treatment modalities. These findings are in contrast with some previous studies reporting higher IVR rates post‐eKSS compared to RNU.^15^, ^16^ A possible explanation is that the majority of RNU patients in our cohort underwent a prior diagnostic URS., which may increase IVR risk due to tumour seeding.^14^ Additionally, we excluded patients with a history of bladder cancer, an independent predictor of IVR after RNU which may contribute to our results.^17^, ^18^ We believe that the exclusion of this confounding group leads to more reliable outcomes. Analysis of all recurrences during follow‐up, rather than just the time to the first IVR, showed that patients post‐eKSS had a consistently higher cumulative risk of IVR compared to those who underwent RNU. Traditional IVR‐FS analyses are event‐based and end when the first recurrence occurs, disregarding any subsequent intravesical recurrences that may happen later in the disease's progression. In contrast, cumulative hazard models account for all recurrence events, offering a more accurate assessment of recurrence burden. The higher cumulative hazard after eKSS may be influenced by more frequent endoscopic surveillance and the organ‐preserving approach. Endoscopic follow‐up may promote intraluminal tumour seeding, potentially explaining for the findings post‐eKSS.^8^

While several studies published on the IVR rate and oncological outcomes post‐eKSS, limited evidence exists regarding the oncological impact of IVR after eKSS.^19^, ^20^, ^21^, ^22^ A recent meta‐analysis highlighted a knowledge gap regarding this specific patient group with significant heterogeneity among studies affecting the reliability of the results.^23^ Therefore, we conducted a survival analysis to assess the OS, CSS and MFS in this patient group. Our findings indicate that IVR does not negatively affect long‐term oncological outcomes, as survival rates post‐eKSS were comparable between patients with and without IVR. The only significant difference observed was in OS post‐RNU. This might be attributable to differences in patient and tumour characteristics since the difference in CSS did not differ.

Interestingly, a discrepancy was observed between the grade of the initial upper tract tumour and subsequent IVR, with several patients presenting with LG‐UTUC developing HG‐IVR during follow‐up. This underlines the importance of thorough surveillance, even in patients with LG disease, as these tumours may still pose a risk of aggressive recurrence. Additionally, initial tumour undergrading, which is reported in up to 30% post‐eKSS, may partially account for the observed shift in tumour grade observed in our cohort.^24^

### 4.1 Strengths and limitations

To our knowledge, this is the first study to analyse survival and oncological outcomes in relation to IVR among UTUC patients without a history of bladder cancer. A major strength is the direct comparison of two treatment modalities within a well‐characterised cohort, enabling evaluation of treatment‐related effects on bladder recurrence, minimising confounding from prior bladder cancer. This study is also the first to use the Andersen–Gill model for recurrent events, providing more reliable outcomes. Overall, our findings support the safety and oncological efficacy of KSS in appropriately selected patients.

Several limitations should be acknowledged. The retrospective single‐centre design may introduce selection bias, and the relatively small sample size limited statistical power, especially for subgroup analyses involving postoperative IVI or dURS intensity. Postoperative IVI protocols changed over time. In the eKSS group, only nine patients received post‐operative IVI as this was implemented in standard local protocol in 2023, whereas IVI after RNU is standard practice for a longer period. The EAU guidelines on UTUC recommend a single IVI prior to foley catheter removal as supported by the ODMIT‐C trial.^10^ However, alternative strategies are being explored including gemcitabine intraoperative^25^ or intraoperative mitomycin C (MMC) in the REBACARE trial^26^ which did not demonstrate efficacy in preventing IVR. Although IVI likely influenced IVR in both RNU and eKSS groups, the cohort size prevented detailed subgroup analysis. Variability in follow‐up schedules may have impacted recurrence detection, and IVR‐FS was evaluated only up to 2 years, limiting evaluation of later events. The lack of molecular data also prevented exploration of tumour clonality or mechanisms of grade progression. Despite these limitations, this study provides meaningful evidence in a field with scarce data and highlights the need for further research. Although IVR was more frequent in the eKSS group, SOs were comparable, indicating that recurrences do not negatively impact long‐term prognosis when managed appropriately. Nevertheless, IVR remains burdensome for patients, highlighting the importance of strategies such as postoperative IVI to reduce recurrence risk and patient burden.

### 4.2 Future perspectives

Future research should prioritise prospective studies to validate these findings, capture late recurrences and assess the impact of diagnostic and surveillance URS on IVR and oncological outcomes. Efforts should focus on reducing IVR incidence and patient burden. Postoperative MMC instillation post‐eKSS, currently under investigation in the SINCERE trial, may lower recurrence rates and decrease procedural and psychological burden^27^ Integration of molecular and genomic analyses may further clarify IVR pathogenesis and identify biomarkers to guide personalised surveillance. In addition, minimally invasive urine‐based assays could enhance early IVR detection, allowing more tailored follow‐up and reducing the need for frequent invasive cystoscopies in low‐risk patients.

## 5 CONCLUSION

Patients with UTUC treated with eKSS have a higher cumulative risk of IVR compared to those undergoing RNU, yet without negative effects on survival or oncological outcomes. Upgrading was observed between primary upper tract tumour and subsequent IVRs. These findings support the continued use of kidney‐sparing strategies in carefully selected patients, provided that strict surveillance and adjuvant measures to reduce IVR are implemented.

## AUTHOR CONTRIBUTIONS

**Orlane J. A. Figaroa:** concept and design, acquisition of data, analysis and interpretation of data, drafting of the manuscript, critical revision of the manuscript, statistical analysis. **Sanne J. Jonker:** concept and design, acquisition of data, analysis and interpretation of data, drafting of the manuscript, critical revision of the manuscript, statistical analysis. **Felix J. Fris:** concept and design, acquisition of data, statistical analysis. **Guido M. Kamphuis:** concept and design, critical revision of the manuscript. **Adriaan D. Bins:** concept and design, critical revision of the manuscript. **R. Jeroen A. van Moorselaar:** concept and design, critical revision of the manuscript. **Joyce Baard:** concept and design, analysis and interpretation of data, critical revision of the manuscript, supervision.

## CONFLICT OF INTEREST STATEMENT

Author J. B. reports speaker fees and consultancy fees from Olympus, Coloplast, Boston Scientific, TSC Life and Storz. All other authors declare no conflicts of interest.

## Supporting information


**Figure S1.** Overview of the relation between the initial UTUC tumour grade and the IVR tumour grade during follow‐up presented in a Sankey‐plot in patients.
