# Supplementary figures and images for: When disease travels downstream: The effect of intravesical recurrences in patients treated for upper tract urothelial carcinoma
Source: BJUI Compass. 2026 Jun 29;7(7):e70225. doi: 10.1002/bco2.70225 (PMC13311729; doi:10.1002/bco2.70225)

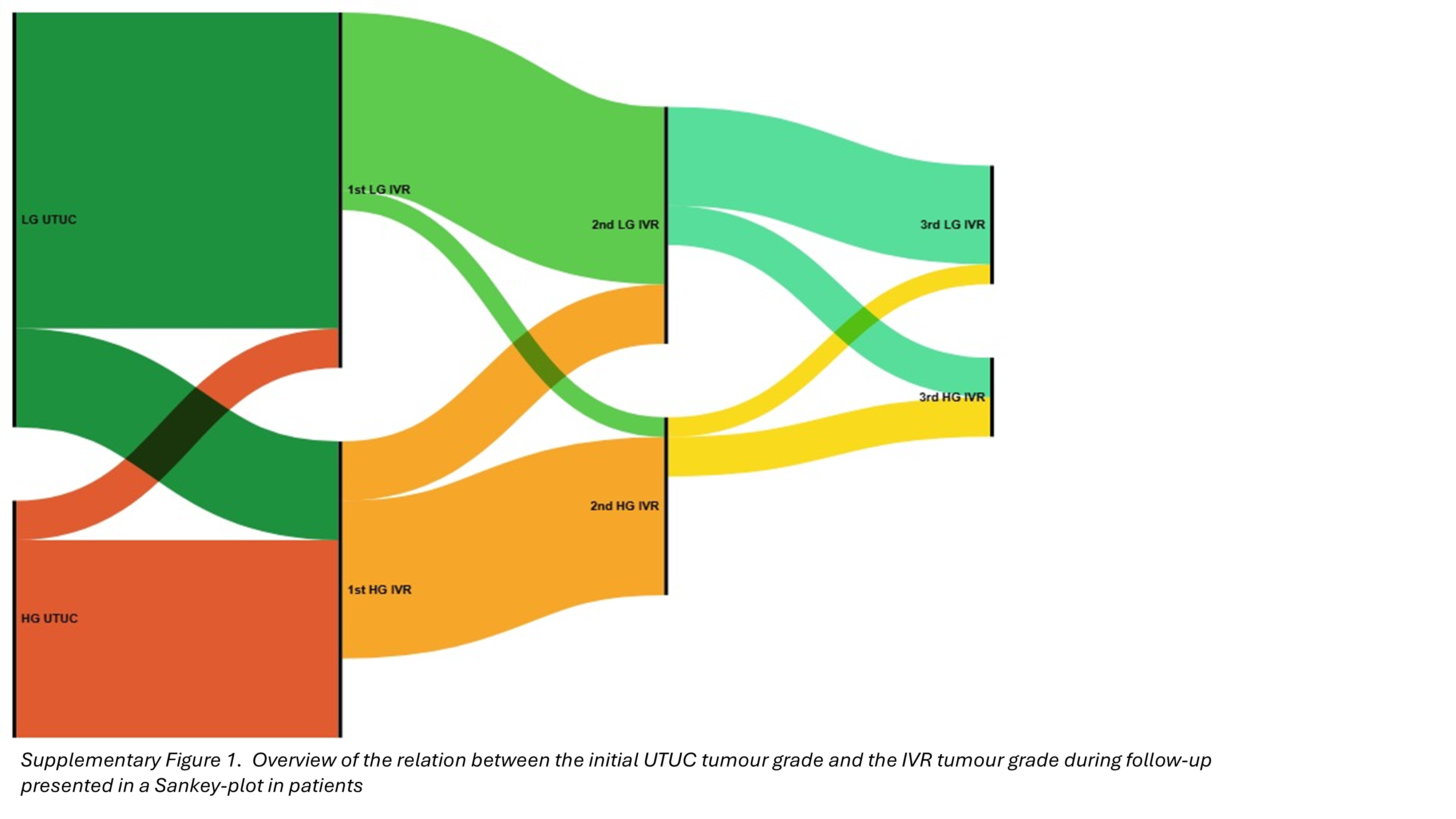

Supplement: Supplementary file 1 — Figure S1. Overview of the relation between the initial UTUC tumour grade and the IVR tumour grade during follow‐up presented in a Sankey‐plot in patients. [file BCO2-7-e70225-s001.png]
